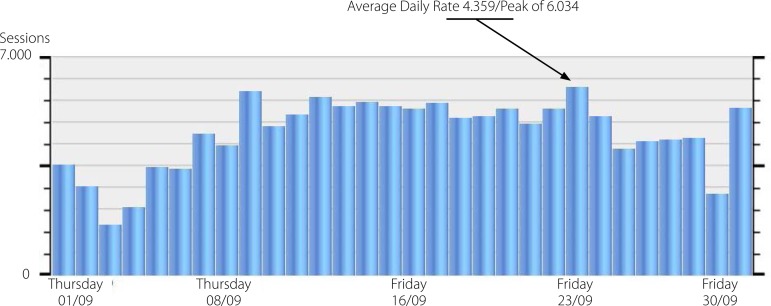# Major Changes with Excellence in Quality

**DOI:** 10.5935/1678-9741.20160071

**Published:** 2016

**Authors:** Domingo M. Braile

**Affiliations:** 1 BJCVS.

We are quickly coming to the third quarter of this complex year 2016. The BJCVS, as you know, has implemented major changes, which will surely bring good results to the visibility of our publication, in order to fulfill its destiny to be the only journal of cardiovascular surgery in the Southern Hemisphere and in Latin America!

However, every change leads to some work and routine modifications required for high quality results. One of the positive changes we had was the increase in number of issues per year, with issues six instead of four. This is a great achievement, but it also increases the need for a quicker editorial process. We have a small but a very efficient staff that strives day and night so we can show that, despite the limitations, we will improve the production logistics.

As it is clear, if we want to remain competitive we must be adherent to the state-of-the-art trends.

Concerning the visibility of our articles, we have implemented, like other international journals, the "Ahead of Print" with a peculiar characteristic, everyone can include the DOI (Digital Object Identifier System).

Thus, articles can be viewed and cited, even if they have not yet been included in a particular issue, and they are also considered as published articles for any eventuality of curriculums updates and also to compose Qualis (CAPES), for instance.

With the advent of Electronic Journals, of which we are part - although we still continue with the print edition - very important changes are taking place, such as the possibility to publish colored figures in high resolution without greater cost for the authors.

In order for us to have figures with high quality, they must be submitted in high resolution with at least 300 DPI (Dots per inch); figures with lower resolution will not be accepted by the submission system.

We also offer the option for authors to include high quality videos in the manuscripts. This is an increasingly trend, as it allows explaining techniques and other details with great precision without the need for long and tiring descriptions.

But be attentive: Videos should preferably be colored and have high resolution. Those produced in white and black, such as echocardiograms and angiograms, will be accepted as originated images.

In this issue, there are 11 articles, including 6 national and 5 international works in the original article, review article, special article and brief communication categories.

On page 337, there is a list of reviewers who provided their time to review manuscripts developing volunteer work, without which it would be impossible to achieve the quality we strive for to make the BJCVS even better.

With our adherence to ScholarOne, the Associated Editors are the ones now who select the reviewers, and I hope they will continue helping us as they did in the last 30 years.

At the moment we have nine Associate Editors, one of them is a Junior Editor, following the global trend of giving opportunity to young people in training period, so we can count on new ideas and create a critical mass that will replace us over time. We admitted Dr. Gabriel Liguori, who is currently in Groningen (the Netherlands), and has already been helping us with the social medias and the editorial process with great interest and knowledge enough to occupy this important position.

The BJCVS is a signatory of the Creative Commons attribution-type BY (CC-BY), which allows your articles to be republished without asking permission, however, the sources must be cited. This is an interesting measure; therefore it increases the distribution opportunities of our name and content of the articles.

This measure was made possible by the collaboration of each of you, SBCCV members and directors, assuming all the expenses of the journal, making it the legitimate representative of a proactive scientific society.

The article "Dexmedetomidine as an Anesthetic Adjuvant in Cardiac Surgery: a Cohort Study," published in the issue 31.3 should be celebrated. It sparked publishers interest in reproducing the article and distribute them to the theme related professionals. We send our congratulations to the authors on this great success!

In these times of change, the word "transparency" has been repeated several times.

We cannot stay out of this necessary trend, which will be greatly facilitated by the metrics offered by ScholarOne which will show all activities related to the performances of the Editor-in-Chief, Associate Editors and Editorial Board. We will soon disclose the statistics that will attest the meritorious work developed by the Journal Staff.

For closing this Editorial, I have excellent news. As you can see below, the consultations of the BJCVS articles had been falling earlier this year, but we reacted with superlatives numbers: average of 4.359 consultations, with more than 6.000 requests in a peak day, showing that we are occupying the national and international space, which we desire and deserve!

I wish you all an excellent reading.

Kind regards,

**Domingo M. Braile** ^1^Editor-in-Chief - BJCVS

## Figures and Tables

**Figure:**